# LncRNAs in Cardiomyocyte Maturation: New Window for Cardiac Regenerative Medicine

**DOI:** 10.3390/ncrna7010020

**Published:** 2021-03-10

**Authors:** Maryam Kay, Bahram M. Soltani

**Affiliations:** Department of Genetics, Faculty of Biological Sciences, Tarbiat Modares University, Tehran 111-14115, Iran; maryam_kay2001@yahoo.com

**Keywords:** cardiomyocyte maturation, lncRNAs, myofibril maturation, regenerative medicine

## Abstract

Cardiomyocyte (CM) maturation, which is characterized by structural, functional, and metabolic specializations, is the last phase of CM development that prepares the cells for efficient and forceful contraction throughout life. Over the past decades, CM maturation has gained increased attention due to the fact that pluripotent stem cell-derived CMs are structurally, transcriptionally, and functionally immature and embryonic-like, which causes a defect in cell replacement therapy. The current challenge is to discover and understand the molecular mechanisms, which control the CM maturation process. Currently, emerging shreds of evidence emphasize the role of long noncoding RNAs (lncRNAs) in regulating different aspects of CM maturation, including myofibril maturation, electrophysiology, and Ca^2+^ handling maturation, metabolic maturation and proliferation to hypertrophy transition. Here, we describe the structural and functional characteristics of mature CMs. Furthermore, this review highlights the lncRNAs as crucial regulators of different aspects in CM maturation, which have the potential to be used for mature CM production. With the current advances in oligonucleotide delivery; lncRNAs may serve as putative therapeutic targets to produce highly mature CMs for research and regenerative medicine.

## 1. Introduction

Directed differentiation of human pluripotent stem cells (hPSCs) to cardiomyocytes (CMs) has achieved remarkable progress over the past decade. These have provided powerful resources to study human development, translational research to discover disease mechanisms, new drug discovery as well as cell replacement therapy [1]. Despite tremendous achievements in producing the mature PSC-derived cardiomyocytes (PSC-CM) through tissue engineering-based methods, the highly mature CMs have yet to be achieved [2,3,4]. Most protocols generate immature CMs, which lack many attributes of adult CMs. Consequently, the generated cells cannot be used for efficient drug screening, modeling of adult-onset disease, and also as a source for the cell replacement therapy [5]. The current challenge is to discover and understand the molecular mechanisms, which control the CM maturation process. Cardiomyocyte maturation is the last phase of heart development characterized by structural, gene expression, metabolic, and functional specializations. Maturation is a complex trait controlled in different aspects by multiple signaling networks and regulators within the cytoplasm and nucleus [6]. In recent years, the advancement of novel technologies like microarray and next-generation sequencing (NGS) has revealed that 98% of noncoding sequences of the genome are actively transcribed and produce noncoding transcripts, including small and long noncoding RNAs (lncRNAs) [7]. The discovery of lncRNAs has expanded a new layer of regulation to the complexity of transcriptome. The emerging pieces of evidence demonstrate the important role of lncRNAs in CM differentiation and maturation [8]. Interestingly, a large number of lncRNAs are cardiac-specific or dynamically expressed during the differentiation and maturation processes. LncRNAs are the most heterogeneous group of regulators that can regulate gene expression through various mechanisms, mainly epigenetic and chromatin regulation mechanisms [9]. In this review, we first discuss the hallmarks of CM maturation following by lncRNAs description. Next, we introduce the most novel and important maturation-related lncRNAs and describe their corresponding molecular mechanism in regulating the CM maturation process.

## 2. Cardiomyocyte Maturation

**Figure 1:**
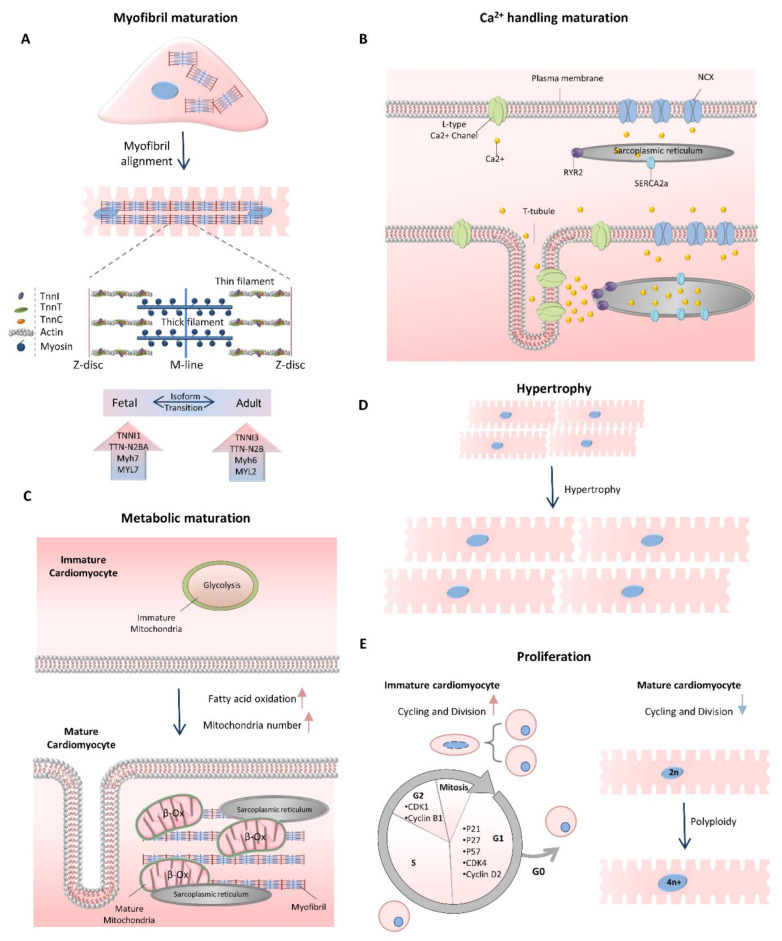
Cardiomyocyte maturation characteristics. (**A**) Cardiomyocytes undergo myofibril maturation, which is followed by increasing the sarcomere length, assembly and expansion; improvement of sarcomere alignment and M-line formation. (**B**) Calcium handling maturation is mediated by the development of T-tubules, expression of calcium handling proteins and increased volume of calcium stores of the sarcoplasmic reticulum. (**C**) Metabolic maturation causes the switch of glycolysis to fatty acid β-oxidation and increases in mitochondria number. The cristae structures in mitochondria are densely organized, and the mitochondria are localized close to the SR and myofibrillar structures to support efficient ATP transition. (**D,E**) Proliferation-to-hypertrophy transition occurs at the time of maturation. The postnatal heart size is increased through maturational hypertrophy followed by polyploidization (top) cardiomyocyte proliferation rate declines through downregulation of cell-cycle-associated genes (bottom).

The dynamic process of cardiac development is a highly orchestrated event that can be divided into three main stages, including specification, morphogenesis, and maturation [6]. A tight gene regulatory network is required to differentiate and specify cardiac lineages from mesodermal progenitor cells [1]. The morphogenesis step is followed by the proper organization of cardiac cells and other structural components of the heart, which is regulated at the molecular, cellular and tissue-level events [10]. At the last step, CM maturation, which is characterized by structural, functional and metabolic specializations, prepares the organ for efficient and forceful contraction and relaxation cycle throughout life [5]. The CM maturation process is an inclusive developmental program that drives a wide range of phenotypic changes, which lead to convert fetal CM to adult CM. The major biological processes involved in CM maturation are myofibril maturation, electrophysiology and Ca^2+^ handling maturation, metabolic maturation and proliferation to hypertrophy transition, which will be described in more detail [5,6]. Myofibrils, which are composed of repeating sections of sarcomeres, are the contractile apparatuses of CMs [11]. Myofibril maturation is characterized by the increase of sarcomere length, assembly and expansion; improvement of sarcomere alignment [12,13] and M-line formation [14], which results in a massive expansion of myofibrils (Figure 1A). The myofibrils organization enhancement is related to sarcomeric isoform switching through either transcriptional changes or alternative splicing [15]. In this process, several sarcomere components switch from a fetal to an adult isoform, which mainly includes *TnnI1* to *TnnI3* [16], *Myh7* to *Myh6* [17], *Ttn-N2BA* to *Ttn-N2B* [18] and *Myl7* to *Myl2* transition [19]. Cardiomyocyte contraction is activated by Ca^2+^ binding to the myofilaments. The electrical impulses and fluctuations of cytoplasmic Ca^2+^ concentration strongly regulate the activity of kinases, phosphatases, exchangers, ion channels, and transporters, which are critical for proper cardiomyocyte contraction [20]. The electrophysiology features of mature cardiomyocytes differ in important ways from immature cardiomyocytes [21]. The increased expression of ventricular ion channels like *Scn5a* [22], *Kcnj12*, *Kcnj2* [23] and Ca^2+^ handling molecules, such as *Cav1.2*, *Ryr2*, and *Serca* [24], resulted in more negative resting membrane potential and faster upstroke velocity of mature cardiomyocytes [25,26]. Furthermore, the mature cardiomyocyte evolves transverse-tubules (T-tubules), which are an extension of cell members penetrating into the center of mature cardiomyocytes and containing large concentrations of ion channels, transporters, and pumps. The presence of T-tubules allows the rapid transmission of the action potential, synchronizing calcium release and regulating cellular calcium concentration (Figure 1B) [27,28,29]. The dramatic alterations in cardiomyocyte metabolism also occur during the maturation process. The well-defined metabolic shift occurred in fetal to neonatal transition in which the primary source of ATP generation changes from glycolysis to fatty acid β-oxidation [30,31]. The mature cardiomyocytes undergo various adaptations to compensate for this demand. This transition is supported by upregulation of metabolic transcriptional regulator likes *Ppagrc1a*, *Para*, *Esrra* and *Nrf1/2* as well as upregulation of fatty acid metabolism and oxidative phosphorylation-related genes [32,33]. The mature cardiomyocytes are highly enriched in mitochondria and occupy up to 40% of cell volume. The cristae structures in mitochondria are densely organized, and the mitochondria are localized close to the SR and myofibrillar structures to support efficient ATP transition (Figure 1C) [34,35,36]. At the time of maturation, the cardiomyocyte proliferation rate declines rapidly through the proliferation-to-hypertrophy transition. This transition is supported through the downregulation of mitogenic signaling pathways. In the mammalian heart NRG1 and its co-receptor ERBB2 as a main players of Neuregulin-ErbB signaling pathway are essential for cardiomyocyte proliferation and can promote the regeneration of cardiac muscle cells [37]. Activation of Notch signaling pathway trigger the BMP10 activity, which in turn promote proliferation of cardiomyocytes through inhibition of the cell cycle inhibitor p57 [38]. It also regulates the transcription of *CCND1* and induces cell cycle reentry and progression in neonatal cardiomyocytes [39]. Hippo signaling pathway is also an important regulator of cardiomyocyte proliferation and heart size [37]. The downregulation of cell-cycle-associated genes like *Cdk1*, *Ccnb1* and *Aurkb*, as well as mentioned proliferation-associated signaling pathways, are the hallmark of this transition [40,41,42,43]. Despite the cell cycle exit, the postnatal heart size also increased more than 30-fold through the maturational hypertrophy [44]. Other aspect of proliferation-to-hypertrophy transition is polyploidization, in which the cardiomyocyte nuclei are polyploid due to DNA endoreplication without karyokinesis (Figure 1D, top) [45,46]. Cardiomyocytes polyploidization also induces the maturational hypertrophy and cause cell cycle exit in mature cardiomyocytes (Figure 1D, bottom) [47]. Finally, efficient integration of cardiomyocytes into cardiac tissues require adhesion like structures and costameres to facilitate extracellular matrix (ECM) attachment as well as intercalated discs (ICDs) structures. ICDs contain fascia adherens, desmosomes, and gap junctions, which support well organized cardiomyocytes junctions and synchronized contraction of cardiac cells [48,49,50,51].

## 3. Noncoding RNAs

The most recent studies indicate that the developmental complexity of higher eukaryotes is raised from the gene regulation process by a large number of regulators rather than the number of protein-coding genes [52]. The protein-coding genes represent only a small portion of the mammalian genome, while the other majority part produces different classes of noncoding RNAs (ncRNAs). Noncoding RNAs as an important hidden layer of regulation have pivotal roles in almost all biological processes. The ncRNAs can be classified based on their length into small (<200 nucleotides) and long noncoding RNAs (>200 nucleotides) [53,54,55].

**Figure 2:**
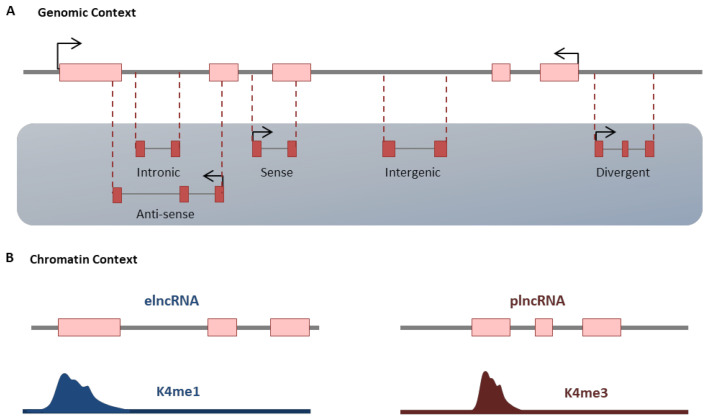
Classification of LncRNA. (**A**) LncRNAs may be divided based on their position and orientation relative to protein-coding genes (genomic context) or by distinct chromatin marks around their transcription start-sites (chromatin context). (**B**) More details are described within the text.

LncRNAs are the most diverse and heterogeneous class of noncoding RNAs [56]. Several transcriptional and post-transcriptional processes, including 5ʹ-capping, splicing and polyadenylation, are done to reach a mature form of lncRNAs [57,58]. Generally, lncRNAs have no open reading frame (ORF), while few studies shed light on the existence of lncRNAs with coding peptides [59]. The lncRNAs have a high degree of tissue specificity [60] and are conserved in either expression pattern or transcript structure despite their low sequence conservation [61,62,63]. The localization of lncRNAs is mainly related to their function. Some of them could be located in both the nucleus and the cytoplasm, while some reside only in the cytoplasm or permanently in the nucleus [64,65]. They could be classified according to their genomic position and orientation relative to their adjacent protein-coding gene [66]. The intergenic lncRNAs (lincRNAs), which do not have any overlap with ding genes, are the most defined group across the organisms [67]. Other groups reside inside the introns of protein-coding genes (intronic lncRNAs), or they are transcribed in the opposite direction of adjacent protein-coding genes (divergent lncRNAs). The lncRNAs can be transcribed from either sense or antisense strand of DNA (Figure 2A) [68,69,70]. They may also be classified based on the chromatin context, in which enhancer-associated (elncRNA) or promoter-associated (plncRNA) lncRNAs are characterized by K4me1 and K4me3 chromatin marks around their transcription start site (Figure 2B) [63]. The critical functions of lncRNAs belong to their ability to interact with regulatory proteins, DNA elements or even functional RNAs to regulate their interaction. LncRNAs can regulate transcription by recruiting transcription factors and chromatin modifier complexes through different mechanisms, including guide, signal, scaffold, or preventing their interaction through decoy mechanism [71,72,73]. LncRNAs also can facilitate the formation of the enhancer-promoter loop at the site of the target locus through regulating inter and intrachromosomal interaction [74,75]. Furthermore the local formation of R-loops (triple-stranded nucleic acid structures with RNA hybridized to duplex DNA) by some anti-sense lncRNAs recruit transcription cofactors to the promoter of corresponded sense mRNA and regulate its transcription [65,76]. The important function of lncRNAs during heart development, differentiation and disease is well recognized. Some important studies revealed the presence of a large number of lncRNAs, which are indispensable for cardiomyocyte maturation (Table 1). The sharp alteration of transcriptome, including lncRNAs at the time of maturation reflects the rapid adaptation of cardiomyocytes for metabolism, regeneration capacity as well as functional properties [8,33]. Below, we review the main lncRNAs involved in the cardiomyocyte maturation procedure.

### 3.1. Mhrt-LncRNA

**Figure 3:**
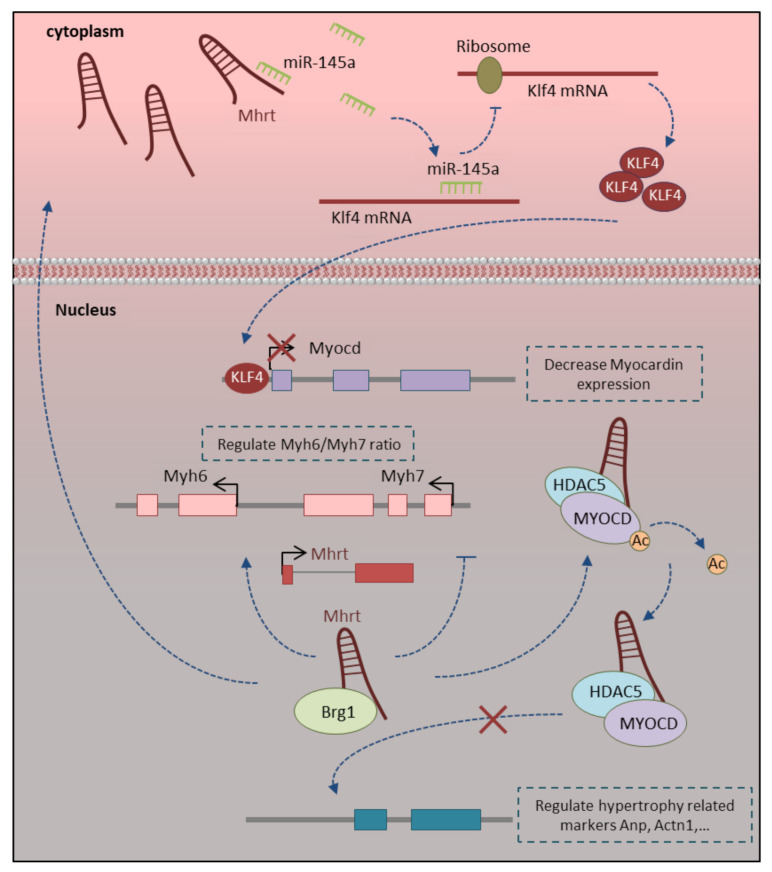
Roles of Mhrt-LncRNA in cardiomyocyte maturation. Mhrt-lncRNA has a different regulatory function in myofibril formation and hypertrophy at the stage of cardiomyocyte maturation. Mhrt regulates the Myh6/Myh7 ratio and their expression during the development through Brg1 inhibition. Cardiomyocyte hypertrophy regulation is also occurred by Mhrt through myocardin expression and function regulation. More details are described within the text.

The Myh-associated RNA transcript (*Mhrt*) is a cardiac-specific lncRNA, which is transcribed within the *Myh* genomic loci. The *Myh* genomic locus not only encodes the main cardiac contractile genes, including *Myh6* and *Myh7* but also miRNAs (*miR-208a* and *miR-208b*) and *Mhrt* lncRNA. *Mhrt* is transcribed in an antisense direction of *Myh6* promoter into the genomic site of *Myh7* [77]. The *Mhrt* expression level is increased gradually from embryonic hearts into mature adult hearts. As a cardiac-specific lncRNA, its expression is limited to the heart with no or minimal expression in other tissues [91]. This lncRNA has a different regulatory function in myofibril formation and hypertrophy at the stage of cardiomyocyte maturation. The lncRNA *Mhrt* has a tight correlation with the *Myh6*/*Myh7* ratio and regulates their expression during the development through *Brg1* inhibition. The BRG1 complex can oppositely regulate the expression of the *Myh6* and *Myh7* contractile genes through the recruitment of different repressors or activators. It can repress the expression of *Myh6* by recruitment of chromatin repressor G9A and DNMT3 on *Myh6* promoter and activate *Myh7* expression through NFAT1 and AP-1 transcription factor recruitment on the site of *Myh7* promoter. *Mhrt* lncRNA directly binds to the helicase domain of BRG1, which causes BRG1 to be sequestered from its target genomic DNA. This negative regulation of BRG1 by *Mhrt* is essential for the normal ratio of *Myh6*/*Myh7* during the development and maturation (Figure 3) [77,116]. On the other side, *Mhrt* regulates cardiomyocyte hypertrophy through myocardin expression and function regulation. In the heart, myocardin, as a strong trans-activator, binds directly to SRF and activates cardiomyocytes restricted genes containing CarG boxes in their promoter region [117]. The *Mhrt* upregulation in the mature heart avoids myocardin expression out of control and protects the heart in the development process. *Mhrt* can raise the *Hdac5* level and enrich the HDAC5/myocardin complex, which leads to myocardin deacetylation. Deacetylation loses the activity of myocardin to regulate the cardiac hypertrophy biomarkers expression (Figure 3) [91]. *Mhrt* lncRNA also inhibits the myocardin expression level through ce-miRNA mechanisms. It sponges *miR-145-5p* in a base-complementary pairing manner, which leads to regulating *Klf4* expression as its target gene. Upregulation of *Klf4* resulted in myocardin downregulation and development of myocardial hypertrophy (Figure 3) [92]. Briefly, *Mhrt* lncRNA have important roles at the time of maturation through regulating the expression of hypertrophy related markers as well as regulating *Myh6*/*Myh7* ratio in the mature heart.

### 3.2. H19-LncRNA

**Figure 4:**
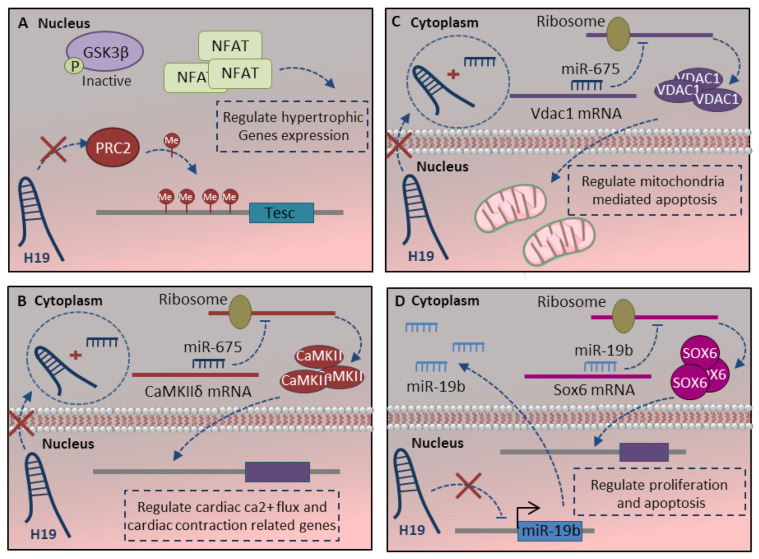
Regulatory effect of H19-LncRNA in cardiomyocyte maturation. (**A**) H19 regulates the cardiomyocyte maturation process at different steps, including apoptosis, proliferation, hypertrophy and contraction. A. H19 physically interacts with PRC2 to suppress pro-hypertrophic NFAT signaling. (**B**) H19 regulates the cardiac ca2+ flux and cardiac contraction function through the H19-miR-675-CamkIIδ axis. (**C**,**D**) The regulatory function of H19 lncRNA in mitochondrial apoptotic pathway and cardiomyocyte proliferation is mediated through either H19-miR-675-Vdac1 (**C**) or H19-miR-19b-Sox6 (**D**) axis. More details are described within the text.

The H19-lncRNA is a highly conserved, imprinted, non-protein-coding gene. The base conservation, along with the highly conserved secondary structure, suggests its pivotal roles in development [96]. H19 has a high-level of expression through cardiomyocyte differentiation, while its expression strongly reduces after birth and through maturation [93]. H19 could regulate the cardiomyocyte maturation process at different steps, including apoptosis, proliferation, hypertrophy and contraction. H19-lncRNA has been demonstrated to regulate hypertrophy in cardiomyocyte cells [80]. H19 physically interacts with polycomb repressive complex 2 (PRC2) to suppress pro-hypertrophic NFAT signaling. Regarding the fact that H19 is downregulated during cardiac development, in the absence of H19, PRC2 hyper-methylates the *TESC* (tescalin) locus as a main inhibitor of NFAT. This leads to activate NFAT signaling and their corresponding hypertrophy-related target genes (Figure 4A) [93]. H19 also delicately regulates the function of proteins associate with cardiac contraction through the H19-miR-675 axis. It is shown that calcium/calmodulin-dependent protein kinase II Delta (*CamkIIδ*) is a direct target of H19-derived *miR-675* (Figure 4B) [80]. *CamkIIδ* is the predominant cardiac isoform, which can phosphorylate ion channels and critical membrane proteins for cardiac electrical activity and structure like L-type Ca^2+^ and voltage-gated Na^+^ and K^+^ channels [118]. The absence of the *H19-miR-675* axis in the mature heart increases *CamkIIδ* expression, which in turn regulates the protein associated with cardiac ca^2+^ flux, cardiac contraction and relaxation.

Other studies also indicate the pivotal role of the *H19-miR-675* axis in the mitochondrial apoptotic pathway. The voltage-dependent anion channel 1 (*Vdac1*) is a direct target of *miR-675*, which is a key protein in the process of mitochondria-mediated apoptosis (Figure 4C) [94]. Moreover, H19-lncRNA regulates proliferation and apoptosis through the *miR-19b-Sox6* axis. H19 inhibits *miR19b* expression, which leads to *Sox6* target gene expression upregulation (Figure 4D). SOX6 is a multifunction transcription factor with critical roles in cell proliferation and apoptosis during the late-stage of cardiomyocyte differentiation [119]. Altogether absence of H19-lncRNA regulates the cardiomyocyte apoptosis, proliferation and protects cardiomyocytes by antiapoptotic effect through either *H19-miR-675* or *H19-miR-19b* axis.

### 3.3. Ahit LncRNA

The antihypertrophic interrelated transcript (*Ahit*) lncRNA was recently reported that regulate hypertrophy in the postnatal heart. *Ahit* is highly enriched in the adult heart and is adjacent to *Mef2a*, which is a pivotal regulator of cardiac development [120]. While the critical function of MEF2 in cardiac development is clear, a novel function of MEF2 transcription factors in the postnatal heart was proposed. The MEF2 expression in the adult heart is at the basal level, and its upregulation to the level seen in the failing heart can promote chamber dilation, mechanical dysfunction, and dilated cardiomyopathy [121,122]. *Ahit* lncRNA regulates chromatin structure and expression of *Mef2a* through direct interaction with SUZ12 (suppressor of Zeste 12) and recruiting PRC2 chromatin remodeler at the promoter region of *Mef2a* locus. PRC2 modulates histone modification and increases H3K27me3 histone marks, which leads to *Mef2a* transcription repression [120]. Altogether, *Ahit* regulates the expression of the *Mef2* family and prevents their abnormal function from inducing precise hypertrophy in mature adult cardiomyocytes.

### 3.4. Zfas1 and Dach1 LncRNAs

**Figure 5:**
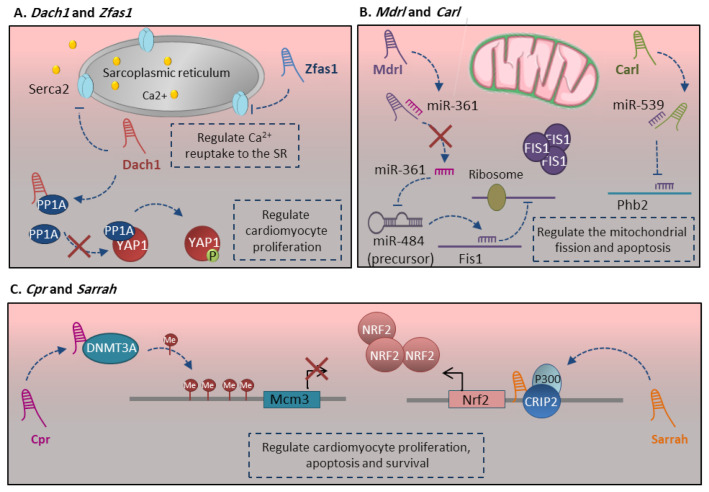
The regulatory function of main lncRNAs related to cardiomyocyte maturation. (**A**) Zfas1 and Dach1 manipulate the cardiac function by regulating Ca2+ homeostasis, proliferation and apoptosis in mature cardiomyocytes. This happened through negative regulation of Serca2a through either its expression repression or functional restriction and regulating the Hippo signaling pathway. (**B**) Carl and Mdrl-lncRNAs regulate mitochondrial fission and apoptosis through regulating the combination of miRNAs and transcription factors in adult cardiomyocytes. Carl lncRNA sponges miR-539 in the cardiomyocytes, which in turn upregulates the expression of Phb2 as its target gene. Mdrl-lncRNA has direct interaction with miR-361 and downregulates its expression levels, which in turn promotes the processing of pri-miR-484 and represses the Fis1 expression. (**C**) Cpr lncRNA attenuates the cardiomyocyte proliferation in postnatal and adult hearts through direct interaction and recruitment of DNMT3A protein Mcm3 promoter to repress its expression. Sarrah lncRNA regulates cardiomyocyte survival and enhances the contractile capacity of adult cardiomyocytes through activating the NRF2 signaling pathway. Sarrah directly binds to the promoter region of the Nrf2 gene through RNA-DNA triple helix formation and recruits CRIP2 and p300 transcription factors to induce its expression. More details are described within the text.

Intracellular Ca^2+^ homeostasis is a key factor to regulate cardiomyocyte contraction as well as cell viability through regulating mitochondrial-mediated apoptosis. This process is tightly regulated by an array of protein and nonprotein-coding genes. SERCA2a (SR Ca^2+^-ATPase 2a) is the main protein, which is indispensable for the normal intracellular Ca^2+^ handling process through regulating the Ca^2+^ reuptake into SR in cardiac muscles [123]. The more recent studies indicate that *Serca2* expression and function are delicately regulated by lncRNAs. *Zfas1* (zinc finger antisense 1), an antisense lncRNA to the 5′ end of the protein-coding gene *Znfx1*, is a cardiac-related lncRNA, which regulates cardiac apoptosis as well as contraction activity [4]. *Zfas1* negatively regulates the *Serca2a* through either its expression repression or functional restriction (Figure 5A). This aims to decrease Ca^2+^ reuptake to the SR, which leads to cytosolic Ca^2+^ overload [81]. The studies indicate that Ca^2+^ overloads cause cardiac dysfunction [81] and induce mitochondria-mediated apoptosis [97].

More recently, a highly conserved lncRNA dachshund homolog 1 (*Dach1*) was also reported by the same group to have a main role in regulating the mature cardiomyocyte proliferation and function. *Dach1* expression was gradually upregulated after birth in a mature heart. It has a hallmark role in the blockage of mitosis and proliferation in the mature heart through the Hippo signaling pathway (Figure 5A) [98]. It is well-known that the Hippo signaling pathway regulates the mitosis and proliferation of cardiomyocytes [124]. *Dach1* directly interacts with protein phosphatase 1 alpha (PP1A) and reduces its dephosphorylation activity. This leads to improving yes-associated protein 1 (YAP1) phosphorylation, reduces its nuclear localization, which in turn inhibits cardiomyocyte proliferation in the mature heart [98]. *Dach1* lncRNA also regulates the function of mature cardiomyocytes through regulating Ca^2+^ homeostasis in the cells. *Dach1* directly interacts with SERCA2a protein, promote its ubiquitination and proteasome degradation (Figure 5A) [82]. All together, *Zfas1* and *Dach1* manipulate the cardiac function by regulating Ca^2+^ homeostasis, proliferation and apoptosis in mature cardiomyocytes.

### 3.5. Carl and Mdrl LncRNAs

Metabolic maturation, which is occurred at the level of prenatal to postnatal transition, is an important event in cardiomyocyte maturation. The increase in myofibril density and contractile components require more ATP production during postnatal hypertrophic growth. Mitochondria are highly dynamic, and their fusion and fission are crucial for maintaining mitochondrial function and fidelity [125]. The mitochondrial biogenesis is tightly regulated by a different network of proteins, miRNAs and lncRNAs. The cardiac apoptosis-related lncRNA (*Carl*), which is highly expressed at the adult heart, suppresses the cardiomyocytes fission and apoptosis by targeting *miR-539* and Prohibitin 2 (PHB2) [88]. PHB2 has main roles in the functional and structural integrity of mitochondria, the cristae morphology and the formation of a super respiratory complex in mitochondria [126]. *Carl* lncRNA sponge *miR-539* in the cardiomyocytes, which in turn upregulates the expression of *Phb2* as its target gene (Figure 5B). PHB2 inhibits the fission in the mitochondria and suppresses apoptosis [88].

The mitochondrial fission and apoptosis are also regulated by other cardiomyocytes enriched lncRNA called mitochondrial dynamic related lncRNA (*Mdrl*). The studies indicate that nucleus enriched *miR-361* inhibits *pri*- to *pre-miR-484* processing through direct interaction with a primary transcript of *miR-484*. *Mdrl* lncRNA has direct interaction with *miR-361* and downregulates its expression levels, which in turn promotes the processing of *pri-miR-484* [89]. Upregulation of *miR-484* represses *Fis1* expression that is involved in mitochondrial fission and apoptosis (Figure 5B) [127]. *Carl* and *Mdrl* lncRNAs regulate mitochondrial fission and apoptosis through regulating the combination of miRNAs and transcription factors in adult cardiomyocytes.

### 3.6. Cpr and Sarrah LncRNAs

Most recent studies indicate the important function of lncRNA *Cpr* (cardiomyocyte proliferation regulator) in mature cardiomyocyte proliferation. The expression level of *Cpr* lncRNA is increased in postnatal and adult hearts and is enriched in the nucleus of cardiomyocytes cell type. This lncRNA attenuates the cardiomyocyte proliferation in postnatal and adult hearts. *Cpr* lncRNA directly interacts and recruits DNMT3A protein to enhance CpG methylation of minichromosomal maintenance 3 (*Mcm3*) promoter (Figure 5C) [78]. MCM3 belongs to the family of proteins involved in the initiation of eukaryotic genome replication and cell cycle progression [128]. The *Mcm3* downregulation in mature cardiomyocytes inhibits cardiomyocyte proliferation. In addition, Liu et al. indicate that *Cpr* remarkably causes a hypertrophic response in mature cardiomyocytes, which has been shown by sarcomere organization, increased cell surface area, and elevated hypertrophy markers like *Anf* and *Myh6* [78].

The association between lncRNAs and cardiomyocyte survival and function is coming to light more by other reports. The lncRNA *Sarrah* (short for SCOT1-antisense RNA regulated during aging in the Heart) was reported to have antiapoptotic and pro-survival effects in adult cardiomyocytes. *Sarrah* directly binds to the promoter region of the *Nrf2* gene through RNA-DNA triple helix formation and recruits CRIP2 and p300 transcription factors (Figure 5C) [99]. The *Nrf2* pathway promotes cell survival and is well-known for its antiapoptotic and cardio-protective effects [129]. The evolutionary conserved lncRNA *Sarrah*, as a regulator of cardiomyocyte survival, also enhance the contractile capacity of adult cardiomyocytes [99].

### 3.7. Other Novel LncRNAs

Most recently, more studies support the critical role of lncRNAs in cardiomyocyte maturation, especially through regulating the hypertrophy, proliferation and conduction system. Terminal differentiation-induced ncRNA (*Tincr*) regulates hypertrophy through direct interaction with EZH2 and regulates the *CaMKII* gene expression [83]. LncRNA *uc.323* also regulates the expression of the cardiac hypertrophy-related gene, carnitine palmitoyltransferase 1b (*Cpt1b*), through direct interaction with EZH2 [109]. The regulation of hypertrophy was also supported by *Magi1-IT1* lncRNA through inactivating Wnt/beta-catenin pathway via targeting the *miR-302e*/DKK1 axis [110]. LncRNA *Snhg1* is a multifunction lncRNA in cardiomyocyte maturation. It can regulate both cardiomyocyte apoptosis by *Snhg1*/*miR-195*/Bcl2l2 [107] or *Snhg1/miR-188-5p*/PTEN [108] axis and cardiomyocytes hypertrophy through *Snhg1*/*miR-15a-5p*/*Hmg1* axis [106]. The cardiac regeneration-associated regulator (*Ecrar*) and *NR_045363* lncRNAs regulate cardiomyocyte proliferation and cell cycle progression through *E2f1-Ecrar*-ERK1/2 [111] and *miR-216a*/JAK2-STAT3 [114] signaling pathways, respectively. Cardiac conduction regulatory RNA (*Ccrr*) [84] and Kcna2 antisense RNA (*Kcna2 AS*) [85] lncRNAs have important roles in regulating the conduction system through regulating the connexin43 (*Cx43*) and *Kcna2* genes expression. These recent studies support the pivotal role of lncRNAs in the maturation process. However, the exact mechanism of their action needs to be elaborated.

## 4. Therapeutic Target of LncRNAs in Cardiac Remodeling

The biological relevance of lncRNAs to different stages of cardiomyocyte maturation has triggered tremendous interest in their application as a therapeutic option. Nevertheless, there are several important issues that need to be considered before lncRNAs can be used as potential therapeutic targets in regeneration medicine. First, many lncRNAs are not conserved at the sequence level, and this makes the identification of human lncRNAs and their clinical testing challenging [130]. Second, as we described in this review, lncRNAs regulate gene expression through different mechanisms, including chromatin remodeling, miRNA regulation or binding to different proteins, RNA and even DNA elements. This phenomenon brings about potential off-target effects for lncRNAs expression, which should be considered. Consequently, the most promising lncRNAs as therapeutic targets are the ones, which their mechanism of action is well described. Finally, the delivery method of oligonucleotides and their duration of action in vivo should be considered before manipulating the candidate lncRNAs [131]. On the other hand, lncRNAs have high tissue specificity, which makes conserved lncRNAs a promising target for drug development, and this also helps to have less remote off-target effects [132]. In spite of the obstacles mentioned above, the role of *CHROME* [133] and *H19* [134] lncRNAs in cardiovascular disease have been investigated in animal models. Of interest, *H19* lncRNA with a pivotal role in the cardiomyocyte maturation process was studied in both mouse and mini-pig models. Li et al. reported that *H19* lncRNA is a novel regulator of smooth muscle cell survival in abdominal aortic aneurysm development and progression [134]. All together, further works should be done to focus and clarify the lncRNAs regulatory network, investigate their role in known beneficial/harmful signaling pathways and optimize the delivery technology in animal models to promote the rapid clinical transformation of lncRNAs. In summary, based on the available in vitro and in vivo animal model, current studies with human cells and tissue and optimized delivery system, lncRNAs has potential to be considered as a novel therapeutic option in different filed of cardiovascular development specially maturation process.

## 5. Conclusions

Over the past decades, several differentiation protocols have been optimized to produce functional cardiomyocytes, which still are structurally, transcriptionally and functionally immature and somehow embryonic-like. In spite of the fact that differences between the immature and mature cardiomyocytes have been explained with details, underlying molecular mechanisms and corresponded regulators that mediate this transition still remain less elaborated. Critical regulators with combinational effects are needed to regulate and optimize cardiomyocyte maturation as a complex process. As previously discussed, several lncRNAs are expressed during the heart development and of interest cardiomyocyte maturation. LncRNAs are defined to regulate the maturation in different aspects, including myofibril maturation, electrophysiology and Ca^2+^ handling maturation, metabolic maturation and proliferation to hypertrophy transition. LncRNAs like *Mhrt*, *H19*, *Zfas1* and *Carl* could act as important participants in promoting the maturation. As a result, they enhance a wide range of phenotypic changes, which promote the fetal to adult cardiomyocytes transition. This review highlights lncRNAs as the multi-effect regulators, which have the potential to promote mature cardiomyocyte production. Targeting the lncRNAs may be a novel approach to producing effective adult-like cardiomyocytes. However, we must consider several challenges, including the lack of sequence conservation, delivery strategy and off-target effect. With the current advances in oligonucleotide delivery and gene therapy, the studies in the next few years may demonstrate the possible role of lncRNAs as a therapeutic target to produce highly mature cardiomyocytes in cell replacement or other clinical therapies.

## Figures and Tables

**Table 1:** LncRNAs with potential roles in the cardiomyocyte maturation process.

Maturation Characteristics	LncRNA	Function	Regulated Target	Ref
**Myofibril formation**	Mhrt	Regulation of Myh6/Myh7 ratio	Brg1	[77]
	Cpr	Sarcomere organization	Mcm3	[78]
	Ppp1r1b	Cardiac myogenesis regulation	Tcap	[79]
**Electrophysiology**	H19	CaMKIIδ expression regulation	miR-675	[80]
	Zfas1	Ca^2+^ homeostasis regulation	Serca2a	[81]
	Dach1	Ca^2+^ homeostasis regulation	Serca2a	[82]
	Tincr	CaMKII expression regulation	EZH2	[83]
	Ccrr	Cardiac conduction regulation	CIP85	[84]
	Kcna2-AS	Cardiac conduction regulation	Kv1.2	[85]
	Malat1	Cardiac conduction regulation	miR-200c	[86]
	ZNF593-AS	Cardiac conduction regulation	HNRNPC	
	Hotair	Regulate Cx43 expression	miR-613	[87]
**Metabolism**	Carl	Regulate metabolic maturation	miR-539	[88]
	Mdrl	Regulate metabolic maturation	miR-361miR-484	[89]
	TTTY15	Regulate mitochondrial energy metabolism	let-7i-5p	[90]
**Proliferation and hypertrophy**	Mhrt	Hypertrophy regulation	Hdac5	[91]
		Hypertrophy regulation/Myocd regulation	miR-145-5p	[92]
	H19	Hypertrophy regulation/NFAT signaling	Prc2	[93]
		Mitochondria-mediated apoptosis regulation by PBX3-dependent way	miR-675 CYP1B1	[94]
				[95]
	Ahit	Hypertrophy regulation/Mef2A regulation	Suz12-Prc2	[96]
	Zfas1	Mitochondria-mediated apoptosis	Serca2a	[97]
	Dach1	Proliferation regulation/Hippo signaling	PP1A	[98]
	Cpr	Proliferation regulation	Mcm3	[78]
	Sarrah	Apoptosis and survival regulation/Nrf2 pathway	CRIP2 and p300	[99]
	Chaer	Hypertrophy regulation	Prc2	[100]
	Meg3	Hypertrophy regulation	miR-361-5p	[101]
		Apoptosis regulation/NF-κB signaling	P53	[102]
	Plscr4	Hypertrophy regulation	miR-214	[103]
	Chast	Hypertrophy regulation	Plekhm1	[104]
	Chrf	Hypertrophy regulation	miR-489	[105]
	Snhg1	Hypertrophy regulation	miR-15a-5p	[106]
		Apoptosis regulation	miR-195	[107]
		Apoptosis regulation	miR-188-5p	[108]
	Uc.323	Hypertrophy regulation	Ezh2	[109]
	Magi1-IT1	Hypertrophy regulation/Wnt signaling	miR-302e	[110]
	Ecrar	Proliferation regulation/ERK1/2 signaling	ERK1/2	[111]
	Crrl	Proliferation regulation/HOPX regulation	miR-199a-3p	[112]
	Uca1	Hypertrophy regulation	miR-184	[113]
	NR_045363	Proliferation regulation/JAK2-STAT3 signaling	miR-216a	[114]
	lncCIRBIL	Apoptosis regulation	Bclaf1	[115]
